# Primary hyperparathyroidism due to atypical vertically long cystic adenoma

**DOI:** 10.1530/EDM-14-0086

**Published:** 2014-10-01

**Authors:** Sachiko-Tsukamoto Kawashima, Takeshi Usui, Yohei Ueda, Maiko-Kakita Kobayashi, Mika Tsuiki, Kanako Tanase-Nakao, Kazutaka Nanba, Tetsuya Tagami, Mitsuhide Naruse, Yoshiki Watanabe, Ryo Asato, Sumiko Kato, Akira Shimatsu

**Affiliations:** 1 Department of Endocrinology and Metabolism, National Hospital Organization Kyoto Medical Center, Kyoto, Japan; 2 Department of Diabetes, Endocrinology and Metabolism, Tango Central Hospital, Kyoto, Japan; 3 Clinical Research Institute, National Hospital Organization Kyoto Medical Center, 1-1 Mukaihata-cho, Fukakusa, Fushimi-ku, Kyoto, 612-8555, Japan; 4 Department of Head and Neck Surgery, National Hospital Organization Kyoto Medical Center, 1-1 Mukaihata-cho, Fukakusa, Fushimi-ku, Kyoto, 612-8555, Japan; 5 Osaka Saiseikai Ibaraki Hospital, Osaka, Japan

## Abstract

Parathyroid cystic adenomas are often misdiagnosed as thyroid cysts and routine preoperative diagnostic tools, such as ultrasonography (US) or 99m technetium-sestamibi (99mTc-MIBI) scans, cannot clearly distinguish between these entities. We present a 67-year-old hypercalcemic woman with a cervical cystic lesion who had negative sestamibi scan results. Her laboratory data indicated primary hyperparathyroidism (serum calcium concentration 14.0 mg/dl, phosphate concentration 2.3 mg/dl, and intact parathyroid hormone (PTH) concentration 239 pg/ml). The cervical US and computed tomography scans revealed a large and vertically long cystic mass (12×11×54 mm). A mass was located from the upper end of the left thyroid lobe to the submandibular region and was not clearly distinguishable from the thyroid. For preoperative definitive diagnosis, we carried out a parathyroid fine-needle aspiration (FNA) and PTH assay (PTH–FNA) of liquid aspirated from the cyst. The intact PTH–FNA concentration was 1.28×10^6^ pg/ml, and the patient was diagnosed with primary hyperparathyroidism due to a cystic mass. She underwent a left upper parathyroidectomy and her serum calcium and intact PTH concentration immediately decreased to normal levels. This report describes the usefulness of PTH–FNA for localizing and differentiating an atypical functional parathyroid lesion from nonfunctional tissue in primary hyperparathyroidism.

**Learning points:**

- Cystic parathyroid lesions, even in the case of elevated PTH levels, can produce negative results in 99mTc-MIBI scans.
- Preoperative diagnosis of parathyroid cysts detectable on US is possible by parathyroid FNA and PTH assay (PTH–FNA) of liquid aspirated from the cyst, if malignancy is not suspected.
- PTH–FNA could be helpful in the differential diagnosis of an equivocal cervical tumor.

## Background

Parathyroid cysts causing primary hyperparathyroidism are uncommon (1) (2). Parathyroid cystic adenomas are often misdiagnosed as thyroid cysts, even in the case of elevated parathyroid hormone (PTH) levels, and especially with asymptomatic hypercalcemia. Routine preoperative diagnostic tools, such as ultrasonography (US) or 99m technetium-sestamibi (99mTc-MIBI) scans, cannot clearly distinguish between these entities (2). We report a hypercalcemic patient with a cervical cystic lesion and negative sestamibi scan results.

## Case presentation

A 67-year-old woman presented to our hospital for further examination of hypercalcemia. Her past medical history was unremarkable, and she had no family history of parathyroid or other endocrine disease. Physical examination revealed a normal blood pressure and a supple neck with no palpable masses or nodules.

## Investigation

**Figure 1:**
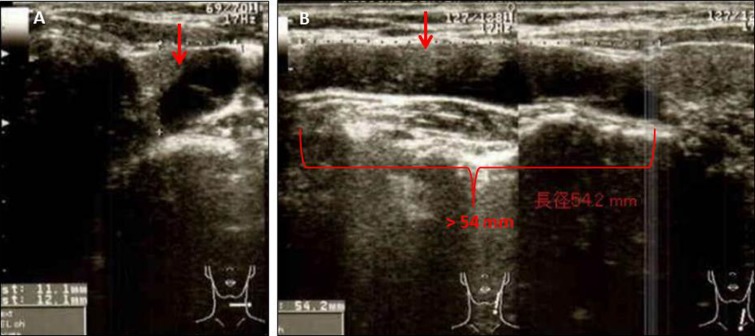
Cervical ultrasonography (US). (A) short axis view and (B) long axis view. Arrow indicates the cystic lesion (12×11×54 mm). The lesion was suggestive of left upper cystic parathyroid adenoma, but not clearly distinguishable from her thyroid gland.

**Figure 2:**
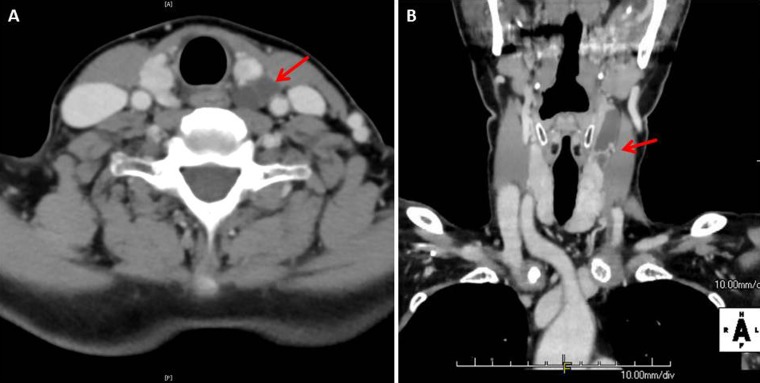
Enhanced computed tomography scan. (A) Horizontal section image and (B) coronal section image. Arrow indicates the cystic lesion with septum.

**Figure 3:**
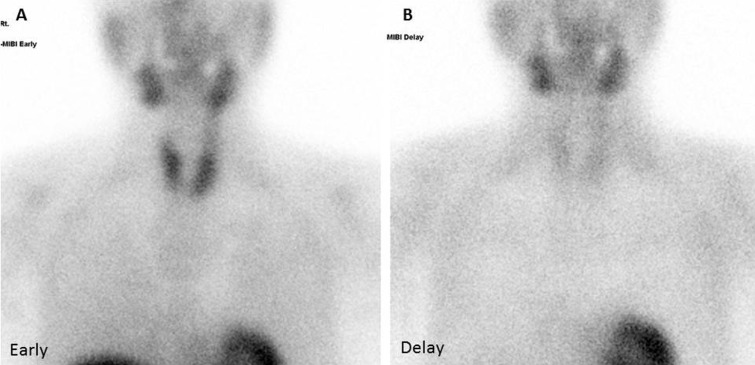
99mTc-MIBI scan. No significant uptake was observed in either the early phase (A) or delayed phase (B).

Laboratory findings revealed an elevated calcium concentration (14.0 mg/dl, normal 8.5–10.2 mg/dl), normal phosphate concentration (2.3 mg/dl, normal 2.3–4.3 mg/dl), and elevated intact PTH concentration (239 pg/ml, normal 15–65 pg/ml). Her other electrolyte values and kidney function were within normal limits. Her 24-h urinary calcium was 260 mg/day, urinary calcium-to-creatinine ratio was 0.51, and fractional excretion of calcium was 2.59%. These findings were compatible with primary hyperparathyroidism. A large and vertically long cystic mass located from the upper end of the left thyroid lobe to the submandibular region (Fig. 1) was detected on performing ultrasonography of the thyroid gland. This finding indicated a left upper cystic parathyroid adenoma, but was not clearly distinguishable from the thyroid even with enhanced computed tomography scan (Fig. 2). The 99mTc-MIBI scanning showed no uptake (Fig. 3). Although the patient was diagnosed with primary hyperparathyroidism, the tumor could not be localized by these imaging studies. For a preoperative definitive diagnosis, we performed a parathyroid fine-needle aspiration (FNA) and PTH assay (PTH–FNA) of the liquid aspirated from the cyst. The aspiration fluid was bloody and cytology revealed no cell components other than hematocytes. The intact PTH–FNA concentration was 1.28×10^6^ pg/ml and the thyroglobulin concentration was 4.0 ng/ml. It was diagnosed that primary parathyroidism was due to a cystic mass.

## Treatment

The patient underwent a left upper parathyroidectomy and her serum calcium and PTH concentrations immediately decreased to normal levels (calcium 8.8 mg/dl and intact PTH 21 pg/ml on postoperative day 1). The histopathologic examination revealed an adenoma of the parathyroid gland with cystic transformation. No findings of malignancy were detected.

## Outcome and follow-up

The hypercalcemic patient had a cervical cystic lesion with negative sestamibi scan results. The cervical US and computed tomography scans revealed a large and vertically long cystic mass of size 12×11×54 mm diameter. A mass was located from the upper end of the left thyroid lobe to the submandibular region, which was not clearly distinguishable from the thyroid. The PTH–FNA assay was performed, and the patient was diagnosed with primary hyperparathyroidism due to a cystic mass. The patient underwent a left upper parathyroidectomy and her serum calcium and intact PTH concentration immediately decreased to normal levels. The usefulness of PTH–FNA for localizing and differentiating an atypical functional parathyroid lesion from nonfunctional tissue in primary hyperparathyroidism is thus evident from the report.

## Discussion

 The imaging studies reveal parathyroid cysts sometimes mimicking thyroid cysts; therefore, preoperative diagnosis of primary hyperparathyroidism due to such cystic lesions is generally difficult. The diagnostic modalities of choice include: US, 99mTc-MIBI scan, computed tomography, and magnetic resonance imaging (2). In some cases, however, the available radiologic methods cannot reliably distinguish between parathyroid or thyroid cysts.

Although the sensitivity of imaging studies to detect solitary functioning parathyroid adenomas is high, some negative results are inevitable. For solitary adenomas, the reported sensitivities of 99mTc-MIBI scans and US are 88.44 and 78.55% respectively (3). One study demonstrated that 99mTc-MIBI scan sensitivity is significantly enhanced by an oxyphil cell predominance within an adenoma (4). The authors reported that sensitivity within the chief and mixed cell-dominant and oxyphil cell-dominant groups was 71.2 and 100% respectively (4). The 99mTc-MIBI may be influenced by negative factors associated with tumor weight, PTH production, cystic lesions, comorbidity with a thyroid lesion, and obesity (5), (6).

A preoperative diagnosis of a parathyroid cyst is possible by FNA of the cyst fluid and PTH determination. PTH–FNA is an effective tool for distinguishing between parathyroid glands and other tissues (7). The limitation of PTH–FNA is an undetected parathyroid adenoma on US. In addition, for parathyroid lesions with suspected malignancy, FNA was not performed to avoid malignant parathyroid cells spreading to other tissues (8). The malignant signs are usually a palpable hard cervical mass, extremely high serum PTH levels, and symptomatic high serum calcium levels (often >14 mg/dl) (9). False-positive results of PTH–FNA have not been reported. In some studies, false-negative results of PTH–FNA were related with aspirate cystic areas within the parathyroid gland or technique failure. Parathyroid aspiration could cause a parathyromatosis and hematoma or adhesion around the gland, making dissection more difficult (2).

The unique finding in this case was the nature of the cyst. The cyst extended upward rather than downward. Parathyroid adenomas generally develop downward. The location of the tumor also differed from that usually predicted for an enlarged parathyroid gland. Parathyroid adenomas tend to locate on the superior dorsal (77%) and inferior dorsal (42%) thyroid gland (10). Because of the atypical features of the cystic lesion, the poor demarcation, and negative 99mTc-MIBI scan results, it was difficult to distinguish between a parathyroid or thyroid cyst. Preoperative PTH–FNA identified lesions that are candidates for surgery. If not contraindicated, PTH–FNA could be helpful in the differential diagnosis of equivocal cervical tumors.

## Patient consent

A written informed consent was obtained from the patient.

## Author contribution statement

S Kato evaluated the patients. S-T Kawashima and T Usui reviewed and performed further investigations on the patient. The ultrasonography-guided fine-needle aspiration was performed by Y Watanabe. Treatment decision was made by all of the authors. Surgery was performed by Y Watanabe and R Asato. The initial draft of the manuscript was prepared by S-T Kawashima. All authors contributed equally to writing the draft and preparing the final manuscript.
